# Nonuniformly-Rotating Ship Refocusing in SAR Imagery Based on the Bilinear Extended Fractional Fourier Transform

**DOI:** 10.3390/s20020550

**Published:** 2020-01-19

**Authors:** Zhenru Pan, Huaitao Fan, Zhimin Zhang

**Affiliations:** 1 Space Microwave Remote Sensing System, Institute of Electronics, Chinese Academy of Sciences, Beijing 100190, China; huaitaofan@163.com (H.F.); zmzhang@mail.ie.ac.cn (Z.Z.); 2 School of Electronic, Electrical and Communication Engineering, University of Chinese Academy of Sciences, Beijing 100039, China

**Keywords:** nonuniformly rotating ships, inverse synthetic aperture radar (ISAR) technique, multicomponent cubic phase signal (CPS), bilinear extended fractional Fourier transform (BEFRFT)

## Abstract

Nonuniformly-rotating ship refocusing is very significant in the marine surveillance of satellite synthetic aperture radar (SAR). The majority of ship imaging algorithms is based on the inverse SAR (ISAR) technique. On the basis of the ISAR technique, several parameter estimation algorithms were proposed for nonuniformly rotating ships. But these algorithms still have problems on cross-terms and noise suppression. In this paper, a refocusing algorithm for nonuniformly rotating ships based on the bilinear extended fractional Fourier transform (BEFRFT) is proposed. The ship signal in a range bin can be modeled as a multicomponent cubic phase signal (CPS) after motion compensation. BEFRFT is a bilinear extension of fractional Fourier transform (FRFT), which can estimate the chirp rates and quadratic chirp rates of CPSs. Furthermore, BEFRFT has excellent performances on cross-terms and noise suppression. The results of simulated data and Gaofen-3 data verify the effectiveness of BEFRFT.

## 1. Introduction

In the marine surveillance of satellite synthetic aperture radar (SAR), nonuniformly-rotating ship refocusing is very significant for the detection and identification of ships. In complex sea conditions, the movements of ships are very complicated. In addition to the self-powered translation, ships also nonuniformly rotate by the influence of sea waves and other factors, which leads to the defocusing of ship images. Many SAR imaging methods [1,2,3,4,5,6] were proposed for moving target refocusing. However, these methods are inapplicable for rotating targets. The inverse SAR (ISAR) algorithm based on the rotatable model has advantages for moving target imaging, especially for rotating targets. Hence, ISAR technique has been widely applied in SAR ship imaging. The range-Doppler (RD) algorithm based on the ISAR technique can be utilized to coarsely focus rotating ship images. The key process of the RD algorithm is motion compensation which includes the range migration and phase compensation. However, due to the time-varying Doppler frequency, nonuniformly rotating ships cannot be finely focused by the RD algorithm. To overcome the Doppler frequency spread, the range-instantaneous Doppler (RID) algorithm utilizes time-frequency transformations [7] instead of Fourier transformations. But this class of algorithms has problems of loss of resolution and cross-terms, which appear as false points in ship images.

In the last decade, many parameter estimation algorithms were proposed in the literature. In [8,9,10,11,12,13], the received signal is modeled as a multicomponent linear frequency modulated (LFM) signal. Radon–Wigner transform (RWT) [9] and Radon-ambiguity transform (RAT) [10] utilize the Radon transform to detect LFM signals. Fractional Fourier transform (FRFT) [13] is also applied to estimate parameters of LFM signals in SAR imaging [5,6]. For gently rotating ships, the above LFM parameter estimation algorithms can be applied.

However, under severe conditions, the rotations of ships could be violent. Therefore, the LFM signal model would no longer be applicable [14,15]. For violently rotating ships, the received signal can be modeled as a multicomponent cubic phase signal (CPS) in [14,15,16,17,18,19,20]. Many cubic phase functions (CPF) [14,15,16,19,20,21,22] were proposed to estimate the parameters of CPSs. In order to reduce cross-terms and enhance auto-terms, the product operation [16] and integrate operation [12,17] were proposed. However, under a rough sea situation with a low signal-to-noise ratio (SNR), the above operations also accumulate noise, which disturbs the detection of auto-terms and causes a bad antinoise performance. The coherent integration was utilized in the coherently integrated generalized cubic phase function (CIGCPF) [19] and the coherently integrated modified cubic phase function (CIMCPF) [20] for a better antinoise performance. With the characteristics of auto-terms parallel to the time axis, the coherent integration utilizes fast Fourier transform (FFT) to separate auto-terms, cross-terms and noise. But the four-order multilinear transformations in CIGCPF and CIMCPF lead to the cross-terms problem, which is also a limitation to antinoise performance.

In this paper, a refocusing algorithm for nonuniformly rotating ships based on the bilinear extended fractional Fourier transform (BEFRFT) is proposed. Different from FRFT, which is a LFM estimator, BEFRFT is a bilinear extension of FRFT and is proposed to estimate the parameters of CPSs. BEFRFT can effectively reduce the disturbance of cross-terms and noise, which is adaptive to low SNR conditions. Combining with RID algorithm, a finely refocused ship image can be obtained.

This paper is organized as follows. Section 2 describes the ISAR imaging model of nonuniformly rotating ships. Section 3 proposes a novel algorithm for the estimation of CPS parameters—BEFRFT—and elaborates on the performances of cross-terms and noise suppression. Section 4 illustrates the implementation procedures of nonuniformly rotating ship refocusing based on BEFRFT. In Section 5, the simulated data and Gaofen-3 data are utilized to illustrate the effectiveness of BEFRFT. Section 6 draws the conclusion of this paper.

## 2. ISAR Imaging Model of the Nonuniformly Rotating Ship

SAR imaging is widely utilized in stationary target imaging. However, it has a limitation for complex moving targets, especially for nonuniformly rotating ships [5,6,23]. Hence, the SAR imaging result of a nonuniformly rotating ship is usually unfocused. As mentioned in Section 1, the ISAR technique can be utilized in SAR images. Before applying the ISAR technique, the inverse azimuth operation (i.e., FFT firstly and then the inverse operation of dechirping) must be utilized to transform the azimuth of SAR image from image domain to time domain.

**Figure 1:**
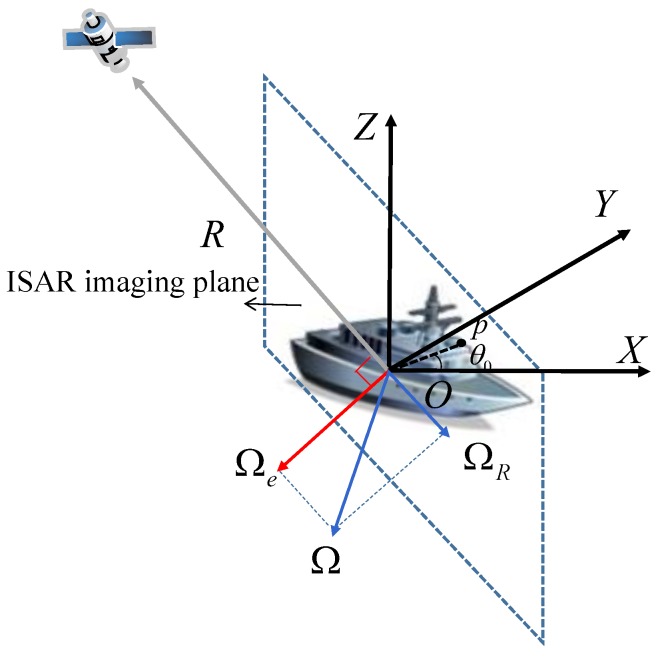
ISAR imaging geometry of a nonuniformly rotating ship.

The ISAR imaging geometry of a nonuniformly rotating ship is shown in Figure 1. The ship is located in the Cartesian coordinate XYZ and nonuniformly rotates around the geometric center *O*. The rotation of ship can be expressed as a synthetic rotation vector $\vec{\Omega }$. The radial direction $\vec{R}$ from the radar to the geometric center *O* is the radar line-of-sight (LOS). $\vec{\Omega }$ can be decomposed into the co-directional component ${\vec{\Omega }}_{R}$ and quadrature component ${\vec{\Omega }}_{e}$. ${\vec{\Omega }}_{e}$ has the only contribution to Doppler effect. The plane viewed from the direction of ${\vec{\Omega }}_{e}$ is the ISAR imaging plane.

Assume that the position of a scattering point *p* is at the distance ${\vec{r}}_{p}$ from the geometric center *O*. The Doppler frequency of *p* can be written as

(1) $$\begin{array}{c} f_{p}=\frac{2}{\lambda }\left(v_{p}+\left({\vec{\Omega }}_{e}\times {\vec{r}}_{p}\right)\cdot \vec{R}\right), \end{array}$$

where λ denotes the wavelength of transmitted radar signal and $v_{p}$ denotes the radial translational velocity between radar and *p*.

Due to the nonuniform rotation of the ship, ${\vec{\Omega }}_{e}$ can be expressed as the Taylor expansion

(2) $$\begin{array}{c} {\vec{\Omega }}_{e}={\vec{\Omega }}_{p}^{\left(0\right)}+{\vec{\Omega }}_{p}^{\left(1\right)}t+\frac{1}{2!}{\vec{\Omega }}_{p}^{\left(2\right)}t^{2}+\frac{1}{3!}{\vec{\Omega }}_{p}^{\left(3\right)}t^{3}+\ldots , \end{array}$$

where ${\vec{\Omega }}_{p}^{\left(n\right)}$ denotes the n-order derivative of ${\vec{\Omega }}_{e}$ and n=0,1,2,3…

After the range migration and phase compensation, the translational velocity can be removed, and the ship signal in a range bin can be written as

(3) $$\begin{array}{c} s\left(t\right)=\sum_{p=1}^{P}\sigma_{p}\exp\left\{j\theta_{0,p}+j\frac{4\pi }{\lambda }\left({\vec{\Omega }}_{p}^{\left(0\right)}t+{\vec{\Omega }}_{p}^{\left(1\right)}t^{2}+\frac{1}{2!}{\vec{\Omega }}_{p}^{\left(2\right)}t^{3}+\ldots \right)\cdot \left({\vec{r}}_{p}\times \vec{R}\right)\right\}, \end{array}$$

where *P* denotes the number of scattering points in a range bin, $\sigma_{p}$ denotes the magnitude of the *p*th scattering point and $\theta_{0,p}$ denotes the initial rotation angle of the *p*th scattering point.

Here, we approximate s(t) as

(4) $$\begin{array}{c} s\left(t\right)\approx \sum_{p=1}^{P}\sigma_{p}\exp\left\{j\theta_{0,p}+j\frac{4\pi }{\lambda }\left({\vec{\Omega }}_{p}^{\left(0\right)}t+{\vec{\Omega }}_{p}^{\left(1\right)}t^{2}+\frac{1}{2!}{\vec{\Omega }}_{p}^{\left(2\right)}t^{3}\right)\cdot \left({\vec{r}}_{p}\times \vec{R}\right)\right\}. \end{array}$$

From Equation (4), we can find that the ship signal in a range bin has the form of a multicomponent CPS. Therefore, we rewrite the ship signal in a general expression as

(5) $$\begin{array}{c} s\left(t\right)=\sum_{p=1}^{P}A_{p}\exp\left[j2\pi (a_{1,p}t+a_{2,p}t^{2}+a_{3,p}t^{3})\right], \end{array}$$

where $A_{p}=\sigma_{p}\exp\left(j\theta_{0,p}\right)$, $a_{1,p}$, $a_{2,p}$ and $a_{3,p}$ denote the center frequency, chirp rate and quadratic chirp rate, respectively.

## 3. Bilinear Extended Fractional Fourier Transform

Fractional Fourier transform (FRFT) [13] is a generalized form of the Fourier transform, which is equivalent to rotating the time axis of the Wigner–Vile plane at an angle and performing a Fourier transformation at zero frequency. LFM signals can be accumulated into straight lines in the Wigner–Vile plane. Hence, FRFT can be utilized to estimate the parameters of LFM signals. However, CPSs are presented as curves in the Wigner–Vile plane, which is inconvenient for estimating their parameters. The bilinear extended FRFT (BEFRFT) is proposed to estimate the parameters of CPSs in Equation (5).

### 3.1. Principle of BEFRFT

Consider a noisy multicomponent CPS.

(6) $$\begin{array}{c} s\left(t\right)=\sum_{p=1}^{P}A_{p}\exp\left[j2\pi (a_{1,p}t+a_{2,p}t^{2}+a_{3,p}t^{3})\right]+n\left(t\right). \end{array}$$

The bilinear correlation function can be written as

(7) $$\begin{array}{cc} R(t,\tau ) & =s\left(t+\tau \right)s\left(t-\tau \right)=R_{auto}\left(t,\tau \right)+R_{cross}\left(t,\tau \right)+R_{noise}\left(t,\tau \right), \end{array}$$

where

(8) $$\begin{array}{c} R_{auto}\left(t,\tau \right)=\sum_{p=1}^{P}s_{p}^{2}\left(t\right)\exp\left[j2\pi \left(2a_{2,p}+6a_{3,p}t\right)\tau^{2}\right] \end{array}$$

denotes the auto-terms; $R_{cross}\left(t,\tau \right)$ and $R_{noise}\left(t,\tau \right)$ denote the cross-terms and noise, respectively.

The cubic phase function (CPF) [12,22] based on NUFFT [24] of $R_{auto}\left(t,\tau \right)$ can be written as

(9) $$\begin{array}{cc} CPF_{auto}\left(t,f_{\tau^{2}}\right) & =\int R_{auto}\left(t,\tau \right)\exp\left(-j2\pi \tau^{2}f_{\tau^{2}}\right)d\tau^{2}\\ \; & =\sum_{p=1}^{P}s_{p}^{2}\left(t\right)\delta \left[f_{\tau^{2}}-\left(2a_{2,p}+6a_{3,p}t\right)\right]. \end{array}$$

We utilize the modulus form to eliminate the influence of $s_{p}^{2}\left(t\right)$ in Equation (9) as

(10) $$\begin{array}{cc} MCPF_{auto}\left(t,f_{\tau^{2}}\right) & =CPF_{auto}\left(t,f_{\tau^{2}}\right)⊙{\left[CPF_{auto}\left(t,f_{\tau^{2}}\right)\right]}^{*}\\ \; & =\sum_{p=1}^{P}A_{p}^{2}\delta \left[f_{\tau^{2}}-\left(2a_{2,p}+6a_{3,p}t\right)\right], \end{array}$$

where ⊙ denotes the Hadamard product and * denotes the complex conjugation.

From Equation (10), we can find that if we rotate the coordinate axis and perform FFT in the direction of $f_{\tau^{2}}=2a_{2,p}+6a_{3,p}t$, the auto-terms can be accumulated at zero frequency and the noise in Equation (7) will spread out over all frequencies. Based on the above statement, the expression of BEFRFT can be written as

(11) $$\begin{array}{cc} BEFRFT(\alpha ,u,f)= & \int MCPF(u\cos\alpha -v\sin\alpha ,u\sin\alpha +v\cos\alpha )\exp(-j2\pi fv)dv, \end{array}$$

where α denotes the rotation angle; *u* and *v* respectively denote the new coordinate axes corresponding to *t* and $f_{\tau^{2}}$; *f* denotes the FFT of *v*.

The BEFRFT of auto-terms can be written as

(12) $$\begin{array}{c} BEFRFT_{auto}\left(\alpha ,u,0\right)=\sum_{p=1}^{P}A_{p}^{2}\int \delta \left[u\left(\sin\alpha -6a_{3,p}\cos\alpha \right)+v\left(\cos\alpha +6a_{3,p}\sin\alpha \right)-2a_{2,p}\right]dv. \end{array}$$

The auto-terms turn into peaks by Equation (12). $a_{2,p}$ and $a_{3,p}$ can be estimated as

(13) $$\begin{array}{cc} ({\hat{a}}_{2,p} & =\frac{u}{2\sin\alpha },{\hat{a}}_{3,p}=-\frac{\cot\alpha }{6})=\underset{\left(\alpha ,u\right)}{\arg\max}\left|BEFRFT\left(\alpha ,u,0\right)\right|. \end{array}$$

### 3.2. Cross-Term Characteristic

Due to the nonlinear transformation, the cross-terms are generated under a multicomponent CPS in Equation (7).

Here, we consider two noise-free CPSs to analyze the characteristic of cross-terms of BEFRFT

(14) $$\begin{array}{cc} s_{12}\left(t\right) & =s_{1}\left(t\right)+s_{2}\left(t\right)\\ \; & =A_{1}\exp\left[j2\pi (a_{1,1}t+a_{2,1}t^{2}+a_{3,1}t^{3})\right]+A_{2}\exp\left[j2\pi (a_{1,2}t+a_{2,2}t^{2}+a_{3,2}t^{3})\right]. \end{array}$$

The auto-terms can be expressed as the form of Equation (8), and the cross-terms can be written as

(15) $$\begin{array}{cc} R_{cross} & \left(t,\tau \right)=s_{1}\left(t+\tau \right)s_{2}\left(t-\tau \right)+s_{2}\left(t+\tau \right)s_{2}\left(t-\tau \right)\\ \; & =2s_{1}\left(t\right)s_{2}\left(t\right)\cos\left\{2\pi \eta \left(t,\tau \right)\right\}\times \exp\left\{j2\pi \left[\left(a_{2,1}+a_{2,2}\right)+\left(a_{3,1}+a_{3,2}\right)t\right]\tau^{2}\right\}, \end{array}$$

where

(16) $$\begin{array}{cc} \eta (t,\tau )=[ & \left(a_{1,1}-a_{1,2}\right)+2\left(a_{2,1}-a_{2,2}\right)t+3\left(a_{3,1}-a_{3,2}\right)t^{2}]\tau +\left(a_{3,1}-a_{3,2}\right)\tau^{3}. \end{array}$$

Obviously, only if η(t,τ)=0 is established, can cross-terms in Equation (15) be accumulated into the form of impulse functions in Equation (9). However, η(t,τ)=0 is hard to be satisfied, especially for real data. Additionally, the following modulus operation and Fourier transform would not generate cross-terms. Hence, BEFRFT is a strict bilinear transformation, which has strong suppression to cross-terms.

Here, we give an example to illustrate the aforementioned content.

**Example** **1.** *Two noise-free CPSs are denoted by Au1 and Au2. The sampling frequency is 256 Hz and the sampling number is 512. The parameters of CPSs are, respectively, as follows: $A_{1}=1$, $a_{1,1}=30$Hz, $a_{2,1}=20$Hz/s, $a_{3,1}=10$$\mathrm{Hz}/s^{2}$ for Au1; $A_{2}=1$, $a_{1,2}=40$Hz, $a_{2,2}=-15$Hz/s, $a_{3,2}=-5$$\mathrm{Hz}/s^{2}$ for Au2.*

**Figure 2:**
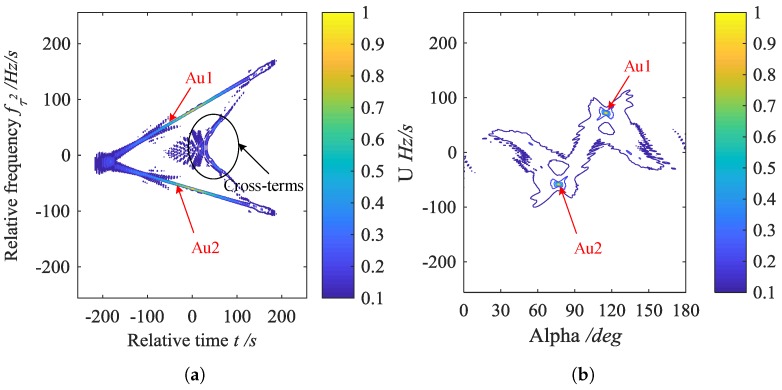

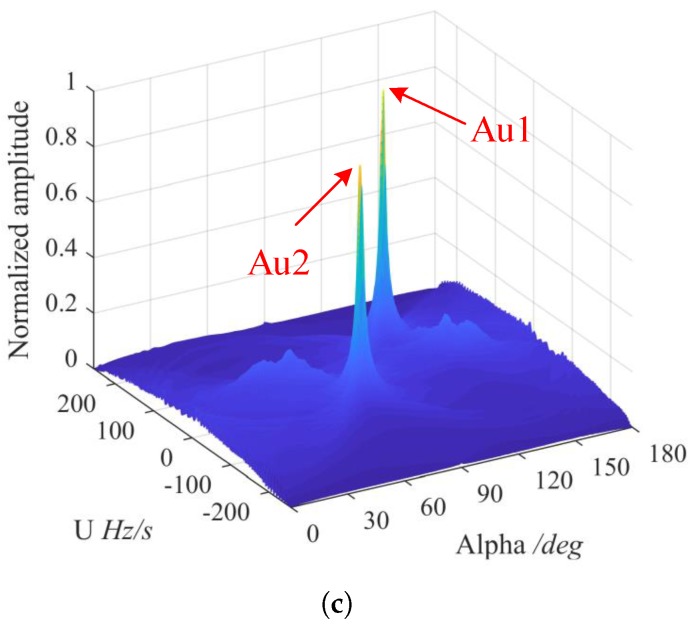
Simulation results. (**a**) Contour of the relative time t–relative frequency $f_{\tau^{2}}$ space of CPF. (**b**) Contour of α–u space of BEFRFT. (**c**) Stereogram of (**b**).

The simulation results are shown in Figure 2. Figure 2a shows the relative time t–relative frequency $f_{\tau^{2}}$ space of CPF in Equation (9). As indicated in Figure 2a, the auto-terms are accumulated into straight lines. However, the cross-terms also exist in a certain form, which increases the difficulty of distinguishing auto-terms. After BEFRFT in Equation (12), it can be seen from Figure 2b,c that the auto-terms are accumulated into peaks and the cross-terms are hardly observed, which means suppression to cross-terms.

In terms of a N-component CPS, the BEFRFT of bilinear transformation generates $\left(N^{2}-N\right)$ cross-terms in Equation (7), while four-order multilinear transformations like CIGCPF and CIMCPF generate $\left(N^{4}-N\right)$ cross-terms. For real data, the generation of cross-terms is greatly reduced by BEFRFT, which can improve the veracity of parameters estimation.

### 3.3. Antinoise Performance

In this subsection, we utilize the input-output SNR [14,18] and mean square error (MSE) [14,15,17,18,21,25] to assess the antinoise performance of BEFRFT. An example is given as follows.

**Example** **2.** *We considered a mono-component CPS with zero-mean white Gaussian noise denoted by Bu. The sampling frequency was 256 Hz and the sampling number was 256. The parameters of Bu were as follows: A=1, $a_{1}=31$Hz, $a_{2}=-23$Hz/s, $a_{3}=10$$\mathrm{Hz}/s^{2}$. The input SNR was $SNR_{in}=\left[-8:1:8\right]$. Two-hundred Monte-Carlo simulations were performed for each input SNR.*

**Figure 3:**
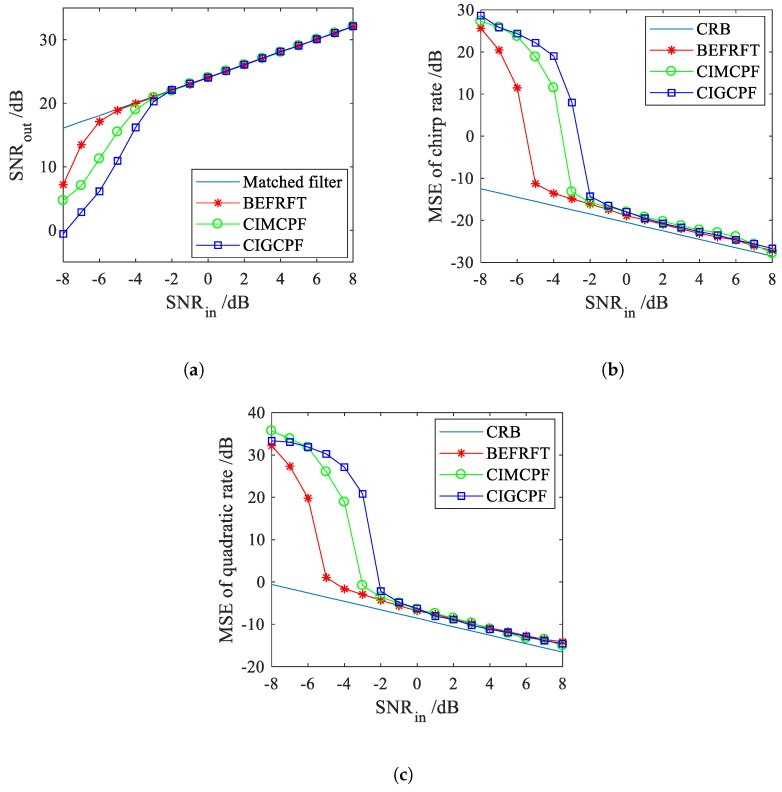
Antinoise performance. (**a**) Input-output signal-to-noise ratio (SNR) comparison. (**b**) Comparison of mean square errors (MSEs) of chirp rate. (**c**) Comparison of MSEs of quadratic chirp rate.

Figure 3a shows the comparison of the input-output SNR of BEFRFT, CIGCPF, CIMCPF and matched filter. When $SNR_{in}\geq -5$ dB, the input-output SNR curve of BEFRFT coincides with the matched filter line, which means the input SNR threshold of BEFRFT is −5 dB. The same as BEFRFT, the input SNR thresholds of CIGCPF and CIMCPF are −2 dB and −3 dB, respectively. We compare the MSEs of chirp rate $a_{2}$ and quadratic chirp rate $a_{3}$ with the Cramer–Rao bounds (CRB) in Figure 3b,c, respectively. Obviously, the input SNR thresholds of BEFRFT, CIGCPF and CIMCPF in Figure 3b,c match the results of Figure 3a. When the input SNR is above the threshold, the MSEs of chirp rate and quadratic chirp rate are close to the CRBs, which indicates the chirp rate and quadratic chirp rate can be estimated accurately.

Hence, we can draw a conclusion that BEFRFT has a better antinoise performance. There are two main reasons: (1) BEFRFT is a bilinear transformation, but CIGCPF and CIMCPF are four-order multilinear transformations. The higher order of transformations lead to the generation of more cross-terms between signal and noise. (2) Unlike the two step estimation of BEFRFT for a CPS, CIGCPF and CIMCPF need three steps, which causes more error propagations.

## 4. Nonuniformly Rotating Ship Refocusing Based on BEFRFT

**Figure 4:**
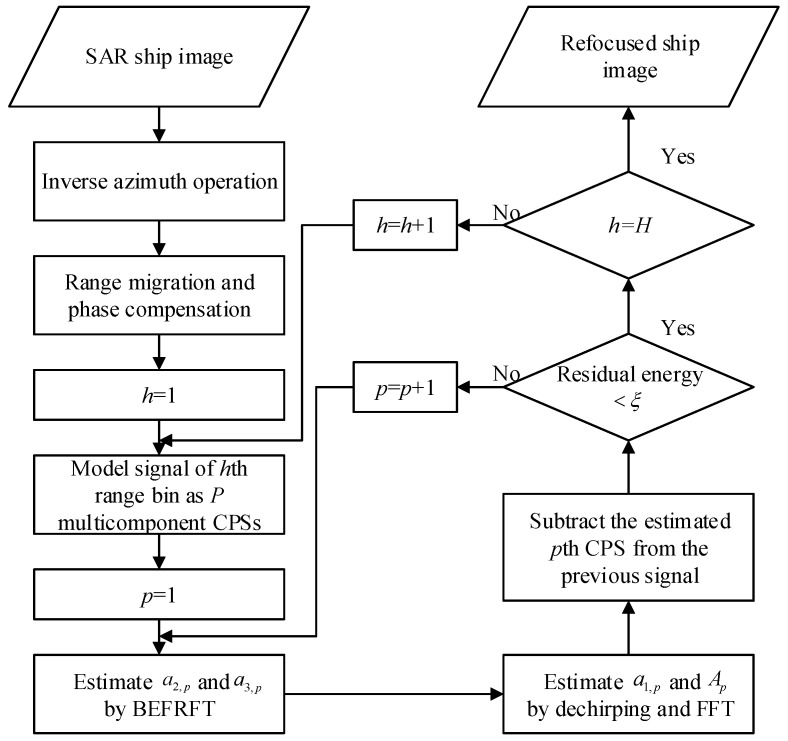
Flowchart of nonuniformly-rotating ship refocusing based on BEFRFT.

The main idea of BEFRFT is estimation of CPS signals’ parameters. Firstly, we utilize BEFRFT to estimate the chirp rate and quadratic rate. Then, we utilize the dechirp technique and FFT to estimate the center frequency and amplitude. The implementation procedures of nonuniformly-rotating ship refocusing based on BEFRFT are illustrated by the flowchart in Figure 4 and described in detail as follows.

**Step 1**  Perform the inverse azimuth operation to the original ship image, as mentioned in Section 2. Apply the range migration and phase compensation to turn the received signals into the turntable form.
**Step 2**  Get the received signal $s_{h}\left(t\right)$ of the *h*th range bin, where 1≤h≤H and *H* is the number of range bins.
  (17) $$\begin{array}{c} s_{h}\left(t\right)=\sum_{p=1}^{P}A_{p}\exp\left[j2\pi (a_{1,p}t+a_{2,p}t^{2}+a_{3,p}t^{3})\right]. \end{array}$$
**Step 3**  Apply BEFRFT to estimate the chirp rate $a_{2,p}$ and quadratic rate $a_{3,p}$.
  (18) $$\begin{array}{c} \left({\hat{a}}_{2,p}=\frac{u}{2\sin\alpha },{\hat{a}}_{3,p}=-\frac{\cot\alpha }{6}\right)=\underset{\left(\alpha ,u\right)}{\arg\max}|BEFRFT\left\{MCPF[s_{h}\left(t\right)]\right\}|. \end{array}$$
**Step 4**  Dechirp $s_{h}\left(t\right)$ with $a_{2,p}$ and $a_{3,p}$ and utilize FFT to estimate the center frequency $a_{1,p}$ and amplitude $A_{p}$.
  (19) $$\begin{array}{cc} ({\hat{A}}_{p}=\frac{D}{N}, & {\hat{a}}_{1,p}=f_{t})=\underset{\left(D,f_{t}\right)}{\arg\max}|FFT\left\{s_{h}\left(t\right)\cdot \exp\left[-j2\pi \left({\hat{a}}_{2,p}t^{2}+{\hat{a}}_{3,p}t^{3}\right)\right]\right\}|, \end{array}$$
  where *D* and $f_{t}$ denote the amplitude and the frequency of the peak after FFT, respectively.
**Step 5**  During the elimination of multicomponent CPSs, the CLEAN technique is utilized [14,15,16,17,19,20]. To prevent the degradation of performance in a low SNR, we subtract each CPS in the frequency domain. The process can be written as
  (20) $$\begin{array}{c} s_{ref}\left(t\right)=\exp\left[-j2\pi \left({\hat{a}}_{2,p}t^{2}+{\hat{a}}_{3,p}t^{3}\right)\right] \end{array}$$
  (21) $$\begin{array}{c} s_{h}\left(t\right)=IFFT\left\{Win\left({\hat{a}}_{1,p}\right)FFT\left[s_{h}\left(t\right)s_{ref}\left(t\right)\right]\right\}, \end{array}$$
  where
  (22) $$\begin{array}{c} Win\left({\hat{a}}_{1,p}\right)=\left\{\begin{array}{cc} 0,\; & f_{L,p}<{\hat{a}}_{1,p}<f_{R,p}\\ 1,\; & otherwise \end{array}\right.. \end{array}$$
**Step 6**  Repeat steps 3–5 until the energy of residual signal is under the energy threshold. The energy threshold ξ can be set to 5% of the original signal energy [14,15]. Then, the estimated ${\hat{s}}_{h}\left(t\right)$ is obtained.
**Step 7**  Repeat steps 2–6 until the received signals of *H* range bins are estimated. Combining RID algorithm, the refocused ship image can be obtained.

## 5. Expeimental Results of Nonuniformly Rotating Ship Refocusing

In this section, the results of ship target simulation are given to illustrate the refocusing performance of proposed BEFRFT, and the Gaofen-3 data are utilized to verify the effectiveness of BEFRFT.

### 5.1. Nonuniformly Rotating Ship Refocusing With Simulated Data

**Figure 5:**
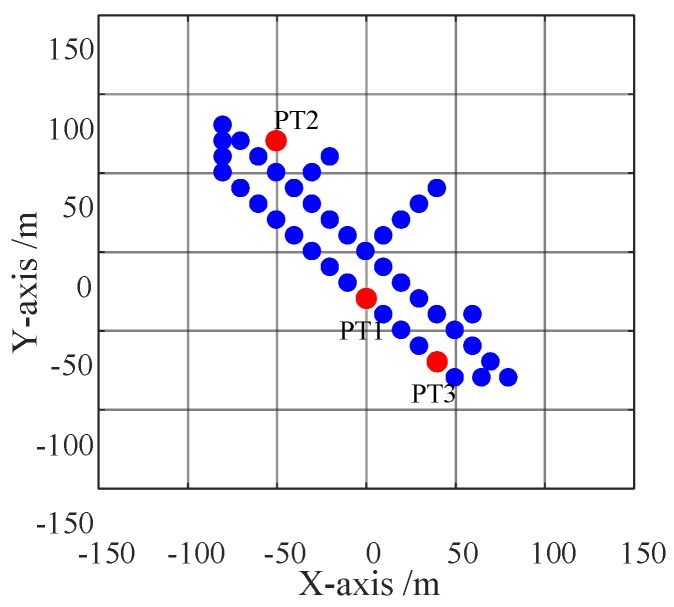
Ship target model.

**Figure 6:**
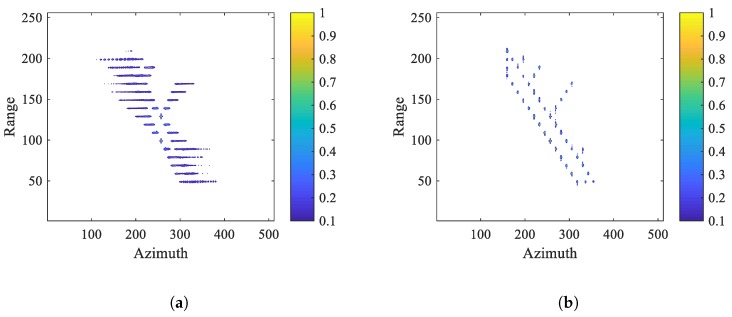
Ship images. (**a**) Ship image based on ISAR algorithm. (**b**) Refocused ship image based on BEFRFT.

**Figure 7:**
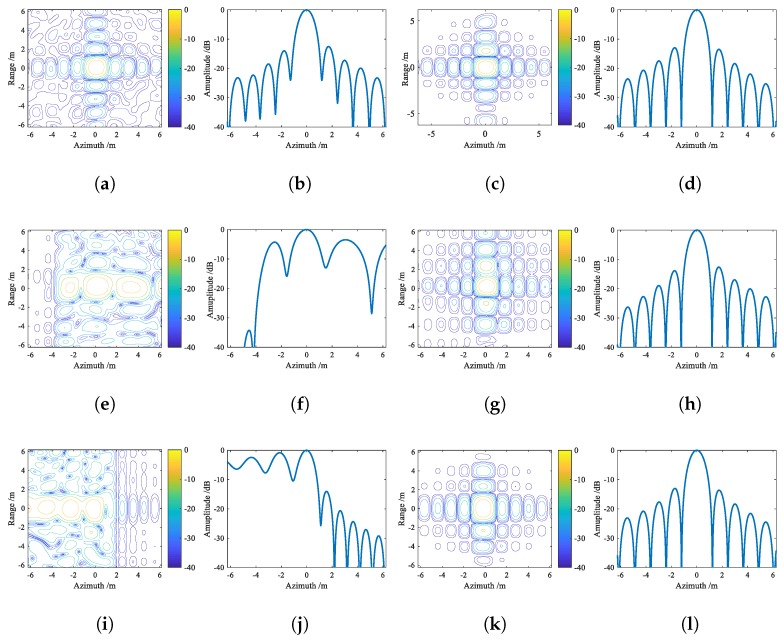
Contour plots and azimuth profiles of PT1, PT2 and PT3. (**a**,**e**,**i**) Contour plots of PT1, PT2 and PT3 based on the ISAR algorithm, respectively. (**b**,**f**,**j**) Azimuth profiles of PT1, PT2 and PT3 based on the ISAR algorithm, respectively. (**c**,**g**,**k**) Contour plots of PT1, PT2 and PT3 based on BEFRFT, respectively. (**d**,**h**,**l**) Azimuth profiles of PT1, PT2 and PT3 based on BEFRFT, respectively.

The parameters of radar system and ship target are listed in Table 1. In Figure 5, the ship target model consists of 42 ideal scatters, and threww representative point targets, PT1, PT2 and PT3, are marked in red. Figure 6 shows ship images in the situation of $SNR_{in}=5$ dB. From Figure 6a, it can be seen the ship image based on ISAR algorithm is blurred in azimuth bin due to the Doppler frequency spread. After applying BEFRFT, the ship in Figure 6b is well-focused. To further illustrate the performance of proposed BEFRFT, contour plots and azimuth profiles of PT1, PT2 and PT3 are given in Figure 7. It can be seen that three point targets are all well-focused after applying BEFRFT.

Peak sidelobe ratio (PSLR) and integral sidelobe ratio (ISLR) are utilized as criteria to assess the quality of refocusing. The imaging quality parameters of PT1, PT2 and PT3 based on ISAR algorithm and BEFRFT are listed in Table 2. It can be seen that the imaging quality parameters of BEFRFT are very close to the theoretical values (i.e., PSLR (−13.26 dB) and ISLR (−9.8 dB)). Both the contour results and imaging quality parameters indicate that the proposed BEFRFT has a good performance on nonuniformly-rotating ship refocusing.

### 5.2. Nonuniformly Rotating Ship Refocusing with the Gaofen-3 Data

**Figure 8:**
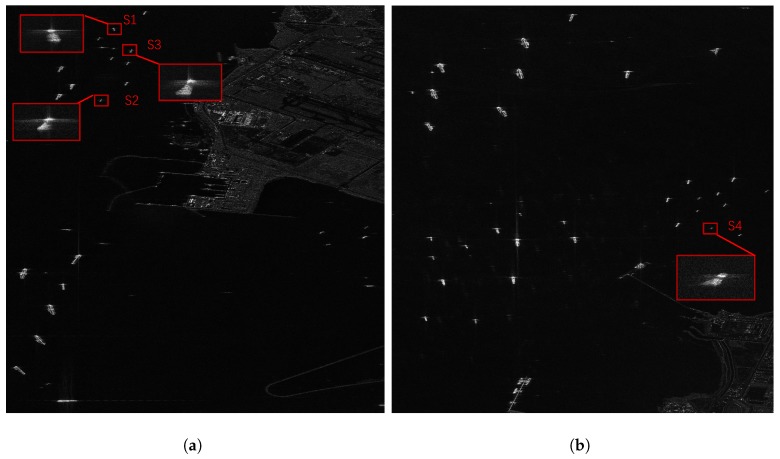
Gaofen-3 images of Singapore. (**a**) Image location: (E104.0, N1.3). (**b**) Image location: (E104.1, N1.3).

Two Gaofen-3 single-look complex (SLC) images of Singapore port were utilized to verify the effectiveness of the proposed BEFRFT, as shown in Figure 8. The latitudes and longitudes of center of images are (E104.0, N1.3) and (E104.1, N1.3), respectively. The Gaofen-3 SAR worked in the sliding spotlight mode and its partial parameters are as follows: radar center frequency $f_{0}$ is 5.4 GHz, the bandwidth *B* is 240 MHz, the pulsewidth $T_{r}$ is 55.0 μs, the pulse repeat frequency is 3125 Hz and the azimuth resolution is 1 m.

From Figure 8, we can find that the majority of ships are relatively big and well-focused, and some of relatively small ships are slightly rotated, which can be refocused by ISAR algorithm. Hence, we selected four small ships, which were nonuniformly rotated, to verify the refocusing performance of BEFRFT. The selected ships, *S*1, *S*2, *S*3 and *S*4, were framed in red and enlarged in Figure 8. The size of ship image slices was 180 m (range) × 176 m (azimuth).

**Figure 9:**
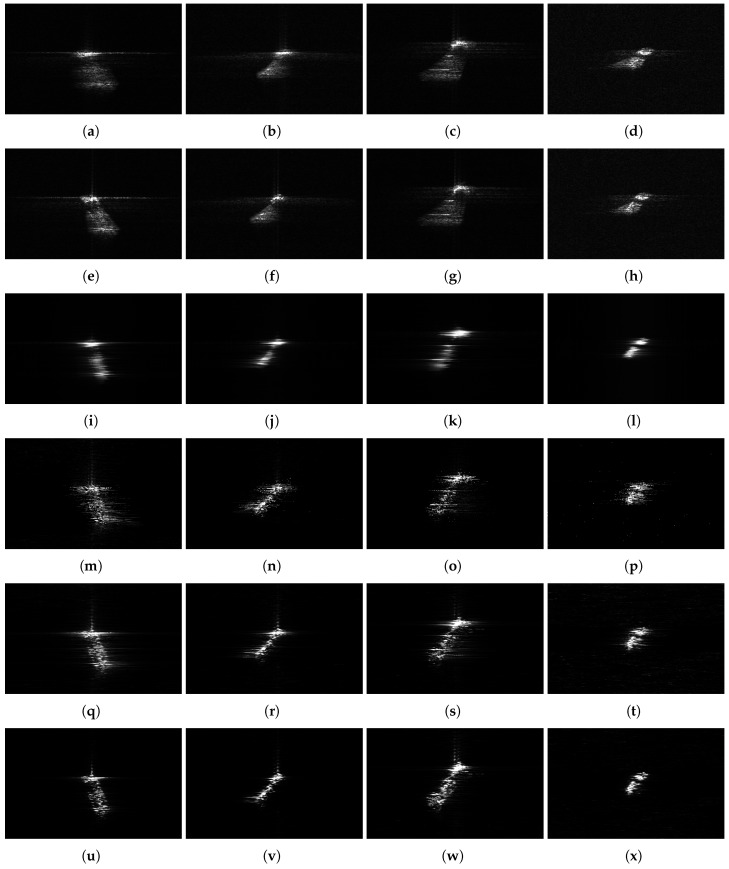
Gaofen-3 ship images of S1, S2, S3 and S4. (**a**–**d**) Original ship images of S1, S2, S3 and S4. (**e**–**h**) Ship images of S1, S2, S3 and S4 based on ISAR algorithm. (**i**–**l**) Refocused ship images of S1, S2, S3 and S4 based on RWT. (**m**–**p**) Refocused ship images of S1, S2, S3 and S4 based on CIGCPF. (**q**–**t**) Refocused ship images of S1, S2, S3 and S4 based on CIMCPF. (**u**–**x**) Refocused ship images of S1, S2, S3 and S4 based on BEFRFT.

Figure 9 shows ship images of *S*1, *S*2, *S*3 and *S*4 based on different methods. As we can see from Figure 9a–d, the original ship images of S1, S2 and S3 are seriously unfocused, and the shape of the ships can hardly be seen. After the inverse azimuth operation and motion compensation mentioned in Section 2, the ship images based on ISAR algorithm are shown in Figure 9e–h. The ship images are still unfocused and the rotation of ships can still be seen. In Figure 9i–l, the classical LFM estimator RWT is utilized to refocus the ship images. The ships are very blurred and the details of ships can hardly be seen. LFM estimators like RWT only estimate the center frequencies and chirp rates of ship signal. Hence, the high-order phase terms cannot be estimated by RWT, which leads to the defocusing in Figure 9i–l. The defocused ship images based on RWT indicate the inadequacy of LFM estimators.

We utilized the CPS estimators to obtain the refocused ship images. Figure 9m–p are the refocused ship images based on CIGCPF. Figure 9q–t are the refocused ship images based on CIMCPF. Figure 9u–x are the refocused ship images based on BEFRFT. Compared to Figure 9m–p and Figure 9q–t—there are less false points around the ships and the details of ships can be seen more clearly in Figure 9u–x.

Here, we utilize the entropy [5,14,15,16,17,19,20] and contrast [5] to assess image quality in Figure 9. An image with a smaller entropy has better image quality. The entropy of an image *I* can be written as

(23) $$\begin{array}{c} E=-\sum_{p=1}^{P}\sum_{h=1}^{H}\frac{{\left|I\left(p,h\right)\right|}^{2}}{S}\ln\frac{{\left|I\left(p,h\right)\right|}^{2}}{S}. \end{array}$$

Contrary to the entropy, a higher contrast means better image quality. The contrast of an image *I* can be written as

(24) $$\begin{array}{c} C=\frac{std(|I\left(p,h\right){|}^{2})}{mean(|I\left(p,h\right){|}^{2})} \end{array}$$

where I(p,h) denotes pixel value at location (p,h), $S=\sum_{p=1}^{P}\sum_{h=1}^{H}{\left|I\left(p,h\right)\right|}^{2}$.

Table 3 shows the entropies and contrasts of ship images of S1, S2, S3 and S4 corresponding to Figure 9. From Table 3, we can find that Figure 9u–x showed the smallest entropies and the highest contrasts, which means better image quality resulted from BEFRFT than from the others. As analyzed in Section 3, BEFRFT has better performances on cross-terms and noise suppression. Hence, BEFRFT has excellent performance on refocusing of nonuniformly rotating ships.

## 6. Conclusions

This paper proposes a refocusing algorithm based on BEFRFT for nonuniformly rotating ships. The received signal is modeled as a multicomponent CPS for each range bin. BEFRFT estimates the chirp rates and quadratic chirp rates of CPSs. Compared with some other algorithms, (1) BEFRFT generates less cross-terms, which reduces the number of false points; (2) BEFRFT has a better antinoise performance for a lower SNR situation. Combining BEFRFT with RID algorithm, the finely refocused ship image can be obtained. Both the simulated data and Gaofen-3 data verify the practicability of proposed algorithm.

## Figures and Tables

**Table 1:** Parameters of radar system and ship target.

Parameters	Values
99825Carrier frequency	10 GHz
Bandwidth	120 MHz
Sampling frequency	150 MHz
Pulse repetition frequency	420 Hz
Range of scene center	5 km
Echo pulses	1024
Translational velocity	40 m/s
Translational acceleration	2 $m/s^{2}$
Translational acceleration rate	1 $m/s^{3}$
Rotational velocity	0.01 rad/s
Rotational acceleration	0.01 $\mathrm{rad}/s^{2}$
Rotational acceleration rate	0.01 $\mathrm{rad}/s^{3}$

**Table 2:** Imaging quality parameters of PT1, PT2 and PT3.

	Target	PSLR (dB)	ISLR (dB)
**ISAR algorithm**	PT1	−12.51	−10.62
	PT2	−3.48	−0.42
	PT3	−0.84	2.98
**BEFRFT**	PT1	−12.95	−10.80
	PT2	−12.64	−10.79
	PT3	−13.82	−10.82

**Table 3:** Entropies and contrasts of ship images.

		Original	ISAR Algorithm	RWT	CIGCPF	CIMCPF	BEFRFT
**Entropy**	S1	6.4257	5.2922	6.1753	5.2798	4.0857	3.5972
	S2	7.8618	7.3830	7.1361	6.7992	5.9165	5.5283
	S3	8.1299	8.5094	7.3393	7.0753	6.5629	6.0525
	S4	8.0640	8.0056	7.2224	6.7422	6.4991	6.1282
**Contrast**	S1	8.8720	9.9634	12.8649	13.5705	13.6443	15.4737
	S2	9.0490	10.0178	13.2321	14.6903	14.8947	16.3034
	S3	9.8960	9.7195	12.4923	14.3366	14.2703	15.7992
	S4	8.5627	9.1625	13.8116	16.6948	16.1005	18.3155
